# Systemic and Mucosal Antibody Responses to SARS-CoV-2 Variant-Specific Prime-and-Boost and Prime-and-Spike Vaccination: A Comparison of Intramuscular and Intranasal Bivalent Vaccine Administration in a Murine Model

**DOI:** 10.3390/vaccines13040351

**Published:** 2025-03-25

**Authors:** Mariam Maltseva, Yannick Galipeau, Pauline McCluskie, Nicolas Castonguay, Curtis L. Cooper, Marc-André Langlois

**Affiliations:** 1 Department of Biochemistry, Microbiology and Immunology, Faculty of Medicine, University of Ottawa, Ottawa, ON K1H 8M5, Canada; 2 The Ottawa Hospital Research Institute, Ottawa, ON K1H 8L6, Canada; 3 Center for Infection, Immunity, and Inflammation (CI3), University of Ottawa, Ottawa, ON K1H 8M5, Canada

**Keywords:** SARS-CoV-2, COVID-19, prime and spike immunization, bivalent vaccines, unadjuvanted intranasal protein vaccine, mucosal vaccines, IgA antibody response, respiratory mucosa, gut mucosa

## Abstract

**Background:** The rapid genetic evolution of SARS-CoV-2 has led to the emergence of immune-evading, highly transmissible variants of concern (VOCs). This prompts the need for next-generation vaccines that elicit robust mucosal immunity in the airways to directly curb viral infection. **Objective:** Here, we investigate the impact of heterologous variant prime–boost regimens on humoral responses, focusing on intramuscular (IM) and intranasal (IN) routes of administration. Using a murine model, we assessed the immunogenicity of unadjuvanted protein boosts with Wu-1, Omicron BA.4/5, or Wu-1 + BA.4/5 spike antigens following monovalent or bivalent IM priming with mRNA-LNP vaccines. **Results:** IM priming induced strong systemic total and neutralizing antibody responses that were further enhanced by IN boosts with BA.4/5. IN boosting achieved the broadest serum neutralization across all VOCs tested. Notably, bivalent mRNA-LNP IM priming induced robust, cross-variant serum neutralizing antibody production, independent of subsequent IN boost combinations. **Conclusions:** Our findings highlight the benefit of including distinct antigenic variants in the prime vaccination followed by a variant-tailored IN boost to elicit both systemic and mucosal variant-specific responses that are potentially capable of reducing SARS-CoV-2 transmission.

## 1. Introduction

The ongoing genetic evolution of severe acute respiratory syndrome coronavirus-2 (SARS-CoV-2) and the emergence of highly transmissible variants of concern (VOCs) have significantly hampered vaccine effectiveness against transmission and symptomatic infection [1,2,3,4,5,6,7,8,9,10,11,12,13,14,15,16,17]. Although booster doses offer protection against severe disease and hospitalization, challenges such as waning immunity, continuous exposure to immune-evasive VOCs, and insufficient induction of mucosal immunity have notably decreased vaccine effectiveness, contributing to the sustained circulation of SARS-CoV-2 [5,6,7,8,9,10,11,17,18,19,20,21,22,23]. The spike protein of SARS-CoV-2 serves as the primary antigenic target of currently approved vaccines, owing to its high immunogenicity, which elicits robust cellular and humoral responses [24,25,26,27,28,29,30]. The substantial antigenic distance between the recently emerged VOCs and the ancestral (Wu-1) SARS-CoV-2 strain, on which the initial COVID-19 vaccines were based, prompts the need to update and tailor vaccines to match the spike proteins of predicted circulating variants, akin to the strategy used in seasonal influenza vaccination.

Following the emergence of Omicron and its sub-variants, vaccine effectiveness and the induction of de novo variant-specific responses has varied after boosting with updated, variant-tailored vaccines [31,32,33,34,35,36,37,38,39,40]. While boosting with these updated vaccines led to a marked increase in neutralizing antibody titers, the highest responses were directed against the ancestral strain, with varying levels of neutralization of recently emerged VOCs, such as Omicron sub-variants XBB.1.5, EG.5.1, and JN.1 [4,20,32,38,41,42,43,44,45,46]. These sub-variants possess mutations in key antigenic sites, enabling them to still evade neutralizing antibodies boosted by updated, variant-tailored vaccines [44,45,47]. This diminished neutralization of current VOCs emphasizes the immune system’s propensity to favor cross-reactive recall responses with greater affinity to the original strain over the generation of de novo antibodies specific to latest exposure, an immunological phenomenon known as immune imprinting [4,20,38,41,42,48,49,50,51,52,53,54]. Thus, boosting strategies that address immune evasion and enhance cross-protection at the site of viral entry are crucial to counter the challenges posed by the continuous emergence of antigenically mismatched variants.

Although intramuscular (IM) vaccination elicits potent systemic cellular and humoral immune responses against respiratory viruses, it leaves vaccine recipients susceptible to infection and viral replication at the primary entry point for SARS-CoV-2, the upper respiratory tract (URT). Mucosal humoral and cellular responses have been shown to correlate with protection against breakthrough infections caused by recently emerged VOCs [22,23,43,55,56,57,58,59]. Several studies have demonstrated superior protection in vivo with oral and intranasal (IN) vaccinations compared to IM vaccination, due to the induction of local immunity in the URT, which limits viral replication and significantly reduces onward transmission of SARS-CoV-2 [22,60,61,62,63,64,65,66,67,68,69,70,71,72,73,74,75,76,77,78,79]. Recent studies have explored several prime–boost approaches, including different vaccine platforms and IN boosting strategies, in the context of pre-existing systemic immunity [22,70,72,73,74,75,79,80]. Notably, Mao et al. showed that an IM prime with an mRNA-LNP vaccine followed by an IN boost with an unadjuvanted SARS-CoV-2 spike protein—a strategy referred to as “Prime and Spike”—leverages pre-existing systemic immunity to induce robust mucosal immune responses without the need for an adjuvant [73]. Collectively, these studies indicate that IN boosting, rather than relying on repeated IM vaccinations, may be a more effective strategy for achieving sustained and potent protection against SARS-CoV-2 reinfection. Despite extensive studies on serum responses to variant-specific boosting, a critical gap remains in understanding how intranasal boosting strategies tailored to circulating variants influence humoral immunity in individuals with pre-existing immunity to an antigenically distant strain. In particular, the effects of heterologous boosting on mucosal variant-specific immunity remain poorly characterized.

In this study, we adapted the “Prime and Spike” approach by Mao et al. to examine the effects of heterologous prime–boost regimens on humoral responses, focusing on different administration routes and variant-tailored vaccine formulations [73]. Understanding the impact of different vaccination strategies on immune responses across different anatomical compartments may aid in inducing protective mucosal responses against SARS-CoV-2 variants. Our findings indicate that the IM prime predominantly shapes the systemic humoral responses, driven primarily by neutralizing antibodies, while the IN boost further amplifies and focuses them. In contrast, only IN vaccination, rather than IM, induced mucosal responses, with variant-specific IN boosts eliciting distinct antigen-specific IgA responses in nasal wash and BALF. Notably, we highlight that bivalent priming with two distinct variants significantly broadened the repertoire of overall neutralizing antibody responses against circulating VOCs at the start, which was further enhanced and focused by variant-specific IN boosting.

## 2. Materials and Methods

### 2.1. Mice Immunization and Sample Collection

All animal experiments were conducted in accordance with the Ontario Animals for Research Act and approved by the University of Ottawa Animal Ethics Committee (protocol BMI-3649). Female, 8-week-old BALB/c mice were obtained from Charles River Laboratories (Saint-Constant, QC, Canada). Prior to each immunization, mice were anesthetized by inhalation of isoflurane in an induction chamber, with the vaporizer set to a 3–5% concentration and an oxygen flow rate of 0.8–1.5 L/min, and vaccinated either intramuscularly or intranasally. Intramuscular vaccinations were administered in the left quadriceps muscle using a 31-gauge syringe, delivering a final dose of 1 μg of Pfizer-Comirnaty monovalent Wuhan (Wu-1) or bivalent (Wu-1 + BA.4/5) vaccine in 20 μL of solution. As described previously in Maltseva et al., intranasal vaccinations involved administering 25 μL per nare, with a total dose of either 1 μg or 10 μg of spike protein formulations consisting of Wu-1, BA.4/5, or Wu-1 + BA.4/5 variants, resuspended in endotoxin-free PBS (Sigma-Aldrich, St. Louis, MO, USA) [66]. SARS-CoV-2 antigens, including full trimeric spike proteins for Wuhan (Wu-1) and Omicron (BA.4/5), were obtained from the Metrology Division of the National Research Council Canada (Montreal, QC, Canada). Briefly, SARS-CoV-2 antigens in prefusion formats were produced in stably transfected CHO (CHOBRI/2353™) cells and purified as described [81]. Control groups included mice vaccinated with phosphate-buffered saline (PBS), either a single- or two-dose regimen of monovalent (Wu-1) or bivalent (Wu-1 + BA.4/5) mRNA vaccine administered IM. Additional groups included mice receiving two IN doses of Wu-1 protein, or a prime–boost regimen consisting of a monovalent (Wu-1) mRNA IM prime followed by a boost with NL63 seasonal human coronavirus spike protein (Creative Diagnostics, Shirley, NY, USA, DAGC133), as summarized in Table 1.

Plasma samples were collected periodically throughout the study via the submandibular vein, with terminal samples obtained by cardiac puncture. Two weeks after the booster dose, plasma, bronchoalveolar lavage fluid (BALF), nasal washes, and intestinal lumen contents were collected to assess mucosal humoral responses, following the method described in Maltseva et al. [66]. At the endpoint of the study, mice were anesthetized via inhalation of isoflurane using an induction chamber, with the vaporizer set to a 3–5% concentration and an oxygen flow rate of 0.8–1.5 L/min. Anesthesia was maintained with a nose cone at the same concentration for euthanasia by cardiac puncture, followed by cervical dislocation. Mucosal samples were collected using PBS supplemented with protease inhibitors (Complete Mini Protease Inhibitor Cocktail tablets, Cat. No.: 11836153001, Basel, Switzerland). A 10 cm section of the small intestine, free of fecal matter, was cut into 1–2 mm pieces and placed in an Eppendorf tube containing 200 μL PBS with protease inhibitors. The sample was incubated on a rocker at 4 °C overnight and centrifuged at 8000× *g* for 10 min at 4 °C, and the supernatants were stored at −80 °C for downstream ELISA analysis. Nasal wash and BALF samples were further concentrated using 50 kDa Pierce Protein Concentrator columns (Thermo Fisher Scientific, Waltham, MA, USA) following the manufacturer’s instructions. Sample volumes were normalized before concentration, and the concentrates were stored at −80 °C until the ELISA assay was performed.

### 2.2. SARS-CoV-2-Specific Antibody Measurements

SARS-CoV-2-specific antibody responses were evaluated as previously described in Maltseva et al. [66]. Briefly, using trimeric spike proteins corresponding to the same variants used for immunizations, spike proteins were diluted to 4 μg/mL in sterile 1X PBS (Multicell Wisent, Saint-Jean-Baptise, QC, Canada, #311-010-CL) and coated onto 384-well immunoplates (ThermoFisher, Waltham, MA, USA, #460372) at 10 μL per well, followed by overnight incubation at 4 °C. Plates were washed three times with 100 μL of PBS-T (PBS + 0.05% Tween-20) to remove unbound antigen and blocked for 1 h at room temperature with shaking using blocking buffer (PBS-T + 3% *w*/*v* non-fat milk powder). Following blocking, serum, BALF, nasal wash, and intestinal supernatant samples were prepared and diluted in dilution buffer (PBS-T + 1% *w*/*v* non-fat milk powder). Serum was diluted 1:300 after the first immunization and 1:500 after the second immunization. BALF samples were diluted between 4- to 30-fold, while nasal wash and intestinal supernatant samples were diluted 4-fold and 2-fold, respectively. After washing the plates three times with PBS-T, 10 μL of the diluted samples was added to the respective wells. The plates were incubated for 2 h at room temperature with shaking and washed three times with PBS-T. Next, 10 μL of the respective secondary-HRP antibody at specified dilutions 1:4000 secondary IgG Anti-Mouse, Human ads-HRP (Southern Biotech, Homewood, AL, USA, #1030-05) and 1:1000 secondary anti-mouse IgA-HRP (Southern Biotech, #1040-05) was added. Plates were incubated for one hour on a shaker, washed thrice with PBS-T, followed by the addition of 10 μL of the SuperSignal ELISA Pico Chemiluminescent Substrate (Thermo Scientific, #37069). Luminescence intensity was measured with BIO-TEK Synergy Neo2 (Agilent, Mississauga, ON, Canada) plate reader for 20 ms/well at a read height of 1.0 mm. Wells containing dilution buffer in place of sample served as negative controls to determine background luminescence. We expressed the SARS-CoV-2-specific antibody levels as relative luminescence values compared to a pooled sample from the Wu-1 + BA.4/5-boosted group, with a pooled sample for each biofluid included as a standard control on every plate. This approach ensured plate-to-plate consistency aligned with results from normalization to the standard IgG and IgA controls (9A9C9 clone) (Genscript, Piscataway, NJ, USA).

### 2.3. Surrogate Neutralization ELISA (snELISA) Assay to Evaluate Neutralization Activity in Serum and BALF Samples

Surrogate neutralization ELISA was performed as previously described in Colwill et al. and Maltseva et al. for the evaluation of the relative inhibition of neutralizing antibodies to spike protein from binding to soluble ACE2 [30,48,81]. Briefly, SARS-CoV-2 proteins, including Wuhan (NRC Metrology), BA4/5 (NRC Metrology), and XBB1.5 (Abwiz Bio, San Diego, CA, USA, 2712), were diluted in sterile 1X PBS to 8 μg/mL and coated onto 384-well Immuno plates (12.5 μL/well) overnight at 4 °C. Plates were washed three times with PBS-T (PBS containing 0.05% Tween-20) and blocked for one hour while shaking. Plasma samples were 1:5 serially diluted in dilution buffer, while BALF samples were diluted 1:3. Diluted samples (20 μL/well) were applied to coated plates and incubated at room temperature for 2 h while shaking. After incubation, plates were washed three times and biotinylated ACE2 (NRC Metrology), diluted to 0.35 ng/μL in a dilution buffer, was added to the wells (20 μL/well). Plates were washed again three times with PBS-T and 20 μL per well of Streptavidin-Peroxidase polymer (Sigma, #S2438) diluted in dilution buffer to 1.25 ng/μL was added. Following one hour incubation at room temperature while shaking, plates were washed thrice with PBS-T and 20 μL per well of freshly prepared SuperSignal ELISA Pico Chemiluminescent Substrate (prepared by mixing a 1:1 ratio of components and further diluted with equal volume dH_2_O) was applied. After 5 min of incubation with shaking, luminescence intensity was measured with a BIO-TEK Synergy Neo2 plate reader for 20 ms/well at a read height of 1.0 mm. To determine the maximum binding signal, control wells were filled with dilution buffer in place of serum, followed by the addition of ACE2 under the same conditions. The relative percentage of inhibition was calculated using the following formula:

$$\%\;Inhibition=(1-\frac{average\;mean\;of\;serum\;sample}{average\;mean\;of\;maxium\;signal})\times 100$$

The serum and BALF dilutions required to achieve 50% inhibition of spike or RBD protein binding to the ACE2 receptor (half-maximal inhibitory dilution, ID50) were determined using a 4-parameter logistic (4PL) curve fitting analysis in GraphPad Prism (9.1.2 software, San Diego, CA, USA). For samples where no neutralization activity was detected, the ID50 value was arbitrarily assigned as half of the lowest dilution tested.

### 2.4. Principal Component Analysis of Neutralization and Serology Data

Principal component analysis (PCA) was performed on normalized serology and neutralization efficiency data from various prime and boost mouse groups. The analysis was conducted using the prcomp function in R 4.2.3, with scaling applied to ensure comparability across variables. Data visualization, including PCA plots and eigenvectors, was carried out using the ggplot2 package. During visualization, samples were annotated based on their prime and boost classifications. Animals with missing values in any measured parameter were excluded from the PCA analysis.

## 3. Results

**Figure 1:**
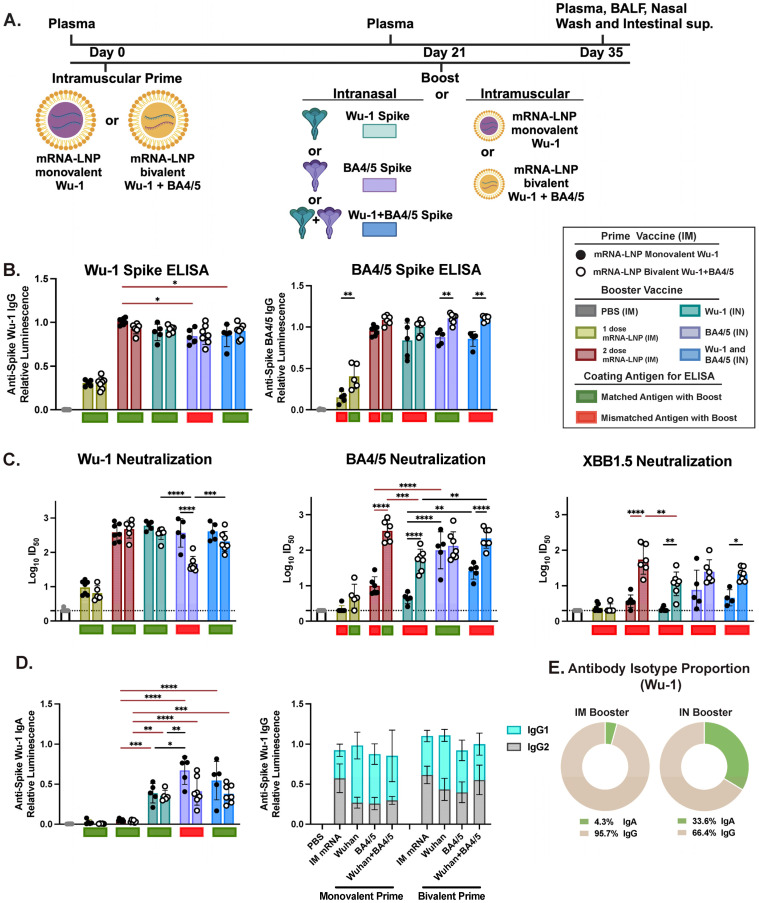
Characterization of systemic immune responses following a heterologous systemic prime and mucosal IN boost vaccination. (**A**) Schematic of the experimental design and vaccine formulations. BALB/c mice were intramuscularly (IM) primed with either a monovalent (Wu-1) or bivalent (Wu-1 + BA.4/5) mRNA vaccine, followed by an intranasal (IN) boost with unadjuvanted Wu-1 spike, BA.4/5 spike, or Wu-1 + BA.4/5 spike on days 0 and 21, respectively. Control groups received PBS, one or two IM mRNA doses, or two IN protein-only doses. Two weeks post-boost, plasma, bronchoalveolar lavage fluid (BALF), nasal wash, and intestines were collected for humoral response analysis. (**B**) Measurement of serum Wu-1 and BA4/5 spike IgG relative titers at endpoint (day 35). (**C**) Reciprocal ID₅₀ neutralization titers based on inhibition of soluble ACE2 binding to immobilized Wu-1, BA.4/5, and XBB.1.5 spike proteins. (**D**) Measurement of Wu-1-spike-specific IgA titers and superimposed IgG1 and IgG2 titers in serum. (**E**) Relative proportion of Wu-1-specific IgG and IgA isotypes in serum. Serum was diluted 1:500 for the measure of IgG-specific titers. Data reflect results from one biological replicate, with values shown as the average of three technical replicates. Antibody titers were log-transformed and analyzed using one-way ANOVA with Tukey’s multiple comparisons test. Unless otherwise stated, all vaccine groups showed significantly higher responses than PBS and one-dose controls and dotted line represents neutralization assay cutoff. Pairwise comparisons were conducted between different booster formulations within monovalent or bivalent prime cohorts, as well as between counterparts with different priming regimens. **** *p* ≤ 0.0001, *** *p* ≤ 0.001, ** *p* ≤ 0.01, * *p* ≤ 0.05.

We set out to assess the effect of heterologous prime and boost vaccine regimens based on different administration routes (IM prime—IM boost vs. IM prime—IN boost) and vaccine formulations (homologous or heterologous prime—boost) on humoral responses in bulb/c mice (Table 1). Mice were initially IM primed with either 1 μg of monovalent mRNA-LNP encoding for SARS-CoV-2 Wu-1 antigen (n = 5 per group) or a bivalent mRNA-LNP encoding for SARS-CoV-2 Wu-1 and BA4/5 antigens (n = 7 per group). After 21 days, mice were boosted with either a second dose of a matching IM mRNA-LNP vaccine or an IN administration of an unadjuvanted Wu-1, BA.4/5, or admixed Wu-1 + BA4/5 formulation of stabilized SARS-CoV-2 spike protein at 1 or 10 μg doses (Figure 1A and Supplementary Figure S3). Control groups received PBS, one dose of IM mRNA-LNP, two doses of unadjuvanted protein via IN only, or 1 dose of IM mRNA followed by IN boost with unadjuvanted NL63 seasonal coronavirus spike protein (Table 1). To evaluate the immunogenicity of the different immunization routes and heterologous prime–boost vaccine formulations, we focused on characterizing IgG and IgA antibody responses in distinct immunological and anatomical compartments. Thus, two weeks post-boost, we collected plasma, bronchoalveolar lavage (BAL), and nasal and intestinal biofluids for a direct comparison of induced antibody profiles following different vaccine regimens (Figure 1A).

Following a single dose of either the monovalent or bivalent mRNA-LNP IM vaccination, all mice exhibited detectable levels of IgG antibodies in serum (Supplementary Figure S1A). IN administration of a 1 μg or 10 μg dose of unadjuvanted protein boosted both IgG and IgA levels in serum, whereas IM administration of the mRNA-LNP vaccine only boosted IgG responses (Figure 1 and Supplementary Figure S3). An IM prime followed by IN boost induced systemic Wu-1-specific IgG levels comparable to those observed after two IM mRNA-LNP doses. However, significantly higher IgA levels were detected in intranasally boosted mice (Figure 1B,D,E), indicating that IN boosting with an unadjuvanted protein can effectively enhance humoral immunity by building upon pre-existing systemic immunity, consistent with previous findings by Mao et al. [73]. Indeed, Wu-1-specific IgG levels were comparable among mice receiving either a homologous or heterologous IN boost (Figure 1B). Notably, we detected significantly higher BA4/5-specific IgG titers in mice primed bivalently and boosted IN with BA4.5 or BA4.5 + Wu-1 antigens compared to their monovalently primed counterparts. A strong correlation (r = 0.84, *p* ≤ 0.001) was observed between BA4/5- and Wu-1-specific IgG antibodies in monovalently primed mice, indicating high antibody cross-reactivity between these antigens. In contrast, no correlation (r = 0.39) was detected in bivalently primed mice, potentially suggesting presence of BA4/5-specific antibody responses (Supplementary Figure S1D). Additionally, although T follicular helper cells were not evaluated in this study, we detected lower ratios of IgG1 over IgG2 in bivalent-primed mice (Figure 1D), suggesting a potential skewing towards a Th1 response [82].

Mao et al. previously reported that unadjuvanted IN boosting with SARS-CoV-1 spike protein, which shares 76% amino acid identity with Wu-1 SARS-CoV-2 spike, leveraged systemic immunity induced by prior vaccination with monovalent Wu-1 mRNA-LNP, and elicited cross-reactive humoral responses [73]. We next sought to determine whether IN boosting with an unadjuvanted NL63 spike, a divergent seasonal coronavirus that also utilizes the ACE2 receptor for cell entry and shares 26% amino acid identity with Wu-1 SARS-CoV-2 spike, could boost and induce cross-reactive humoral responses. However, we found that IgG levels were comparable to those observed in mice after a single dose of IM mRNA-LNP, with no detectable NL63-specific IgG responses (Supplementary Figure S1B). This finding highlights the specificity of memory responses and the importance of using a more closely matched boosting antigen to leverage systemic immunity induced by prior vaccination. This experiment clearly shows that the IM mRNA-LNP prime response did not produce antibodies, or cross-reactive antibodies that could be boosted by an unadjuvanted divergent protein boost.

### 3.1. IM Prime Shapes Systemic Humoral Responses, Primarily Driven by Variant-Specific Neutralizing Responses

Although some statistically significant differences were observed, overall, total antibody responses showed minimal variation among the different vaccination groups (Figure 1B). We next assessed neutralizing activity using a surrogate neutralization ELISA assay (snELISA) to evaluate the breadth of antibody responses from heterologous prime–boost formulations and administration routes (Figure 1C; Supplementary Figure S3B), as systemic neutralizing responses are a well-established correlate of vaccine efficacy [83,84]. The snELISA assay we used measures the relative inhibition of spike to ACE2 receptor binding, as previously described by our group and shown to quantitatively correlate with pseudotyped or infectious virus neutralization assays [85].

Spike binding inhibition for a specific variant was generally superior in mice boosted with the corresponding matched antigen, regardless of whether the boost was administered IN or IM (Figure 1C; Supplementary Figure S3B). In line with the serological data, we observed comparable Wu-1 spike inhibition in mice that were boosted via IM or IN, except those primed with the bivalent mRNA-LNP vaccine and boosted with BA4/5 antigen (mismatched), likely due to significantly lower levels of Wu-1 RBD-specific IgG antibody (Supplementary Figure S1A). In contrast, a significant reduction in BA4/5 spike neutralization was observed in monovalently primed and Wu-1 (mismatched)-boosted mice compared to those boosted with BA4/5 antigen (matched) or their bivalently primed counterparts (Figure 1C; Supplementary Figure S3B). Furthermore, boosting with Wu-1 antigen failed to elicit any neutralizing activity against XBB1.5 spike, while boosting with the more closely related BA4/5 antigen enhanced neutralization.

**Figure 2:**
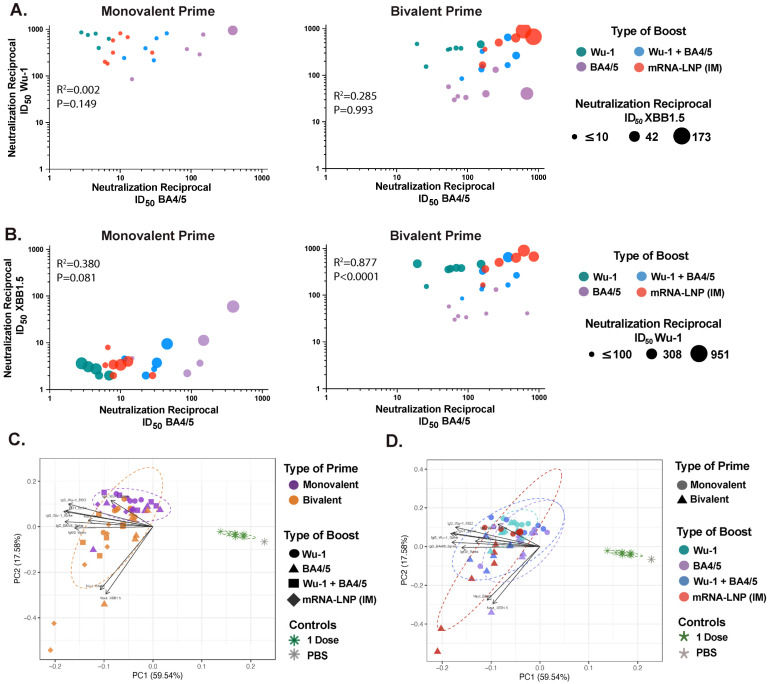
Prime vaccination drives distinct humoral (total and neutralizing) responses in serum, while booster vaccination further refines these responses. (**A**) Correlation between BA4/5 and Wu-1 neutralization (ID_50_) reciprocal titers, with data points colored by booster type. Point size reflects neutralization potency against the XBB.1.5 variant. (**B**) Correlation between BA.4/5 and XBB.1.5 ID₅₀ titers, with data points colored by booster type. Point size reflects neutralization potency against the Wu-1 variant. (**C**,**D**) Principal component analysis (PCA) of serum antibody responses. Each point represents an individual mouse, incorporating all antibody response measurements from Figure 1 and dotted ellipses represent confidence interval for each group. PCA plots are colored by (i) prime vaccine type and (ii) booster type to illustrate distinct clustering patterns elicited by different vaccination regimens.

Notably, IN boost with BA4/5 antigen (matched) induced significantly superior BA4/5 spike neutralization and, although not statistically significant, enhanced neutralization against XBB1.5 compared to mice receiving monovalent mRNA-LNP vaccine IM boost. However, boosting with either Wu-1 or BA4/5 antigens resulted in skewed neutralizing response specific to the boosting antigen (Figure 1C and Figure 2A). Indeed, Wu-1 homologous prime–boost vaccination resulted in significant decrease in neutralization activity against antigenically distant SARS-CoV-2 variants (Figure 1C and Figure 2A,B). This effect was mitigated by heterologous boosting with the BA4/5 antigen, which likely elicited cross-reactive or de novo BA4/5-specific responses given the maintained inhibition of Wu-1 spike but markedly improved neutralization against BA4/5 spike (Figure 1C and Figure 2A,B). Interestingly, we found that BA4/5 + Wuhan boost elicited comparable or superior spike neutralization across all three variants evaluated in bivalent-primed mice, relative to matched or mismatched variant-specific boosts (Figure 1C and Figure 2A,B). In contrast, mice that were primed with the monovalent Wu-1 mRNA-LNP vaccine and boosted with the BA4/5 + Wuhan vaccine exhibited diminished neutralization of BA4/5 and XBB1.5. This suggests that the BA4/5 + Wuhan boost led to the induction of cross-reactive recall responses to epitopes shared between the primary (Wu-1) and secondary (Wu-1 and BA4/5) exposures, rather than generating BA4/5-specific de novo antibodies, indicating an immune imprinting response.

### 3.2. Bivalent mRNA-LNP IM Prime Broadens Neutralizing Responses in Serum

Overall, we found a positive correlation between spike neutralization activity and Wu-1- or BA4/5-specific IgG antibody titers when the boosting antigen matched with the variant used in the snELISA (Supplementary Figure S1C). Importantly, significantly superior BA4/5 and XBB1.5 spike neutralization was noted in bivalent-primed mice. Indeed, we observed a strong positive correlation (Spearman r = 0.877, *p* ≤ 0.0001) between BA4/5 and XBB1.5 spike neutralization in bivalently primed mice compared to monovalently primed groups (Spearman r = 0.380, *p* = 0.081) (Figure 2B). Next, we performed a principal components analysis (PCA) which included paired total and neutralizing responses across the various prime and boost groups (Figure 2C,D). The first two principal components explained 77% of the variation in the data. By coloring the data points according to the type of prime or boost vaccine formulation, we were able to visualize the relative influence of each vaccine formulation on the overall humoral response outcome. Notably, we observed differential clustering based on the type of prime rather than the boost, which was primarily driven by variant-specific neutralizing responses (Figure 2C,D).

Following an IN boost with a 1 μg dose of unadjuvanted protein, similar patterns in variant-specific systemic responses emerged where groups boosted with the corresponding (matched) antigen generally developed the greatest reactivity and variant-specific neutralizing responses (Supplementary Figure S3A,B). Notably, the Wu-1 homologous prime–boost regimen led to Wu-1-skewed total and neutralizing responses while bivalently primed mice exhibited significantly greater total and neutralizing responses (Supplementary Figure S3E). Indeed, although 1 μg IN-boosted mice grouped separately in the PCA, the bivalently primed mice clustered more closely with the 10 μg-boosted groups (Supplementary Figure S4A,B). Taken together, these results suggest that bivalent priming expands the breadth of neutralizing responses beyond what can be elicited with variant-specific boosting. Therefore, our data suggest that the prime vaccine shapes the outcome of total and neutralizing responses in serum, while the booster further refines these responses.

### 3.3. Variant-Specific IN Vaccines Induce Distinct Antigen-Specific IgA Responses in Nasal and BAL Biofluids

**Figure 3:**
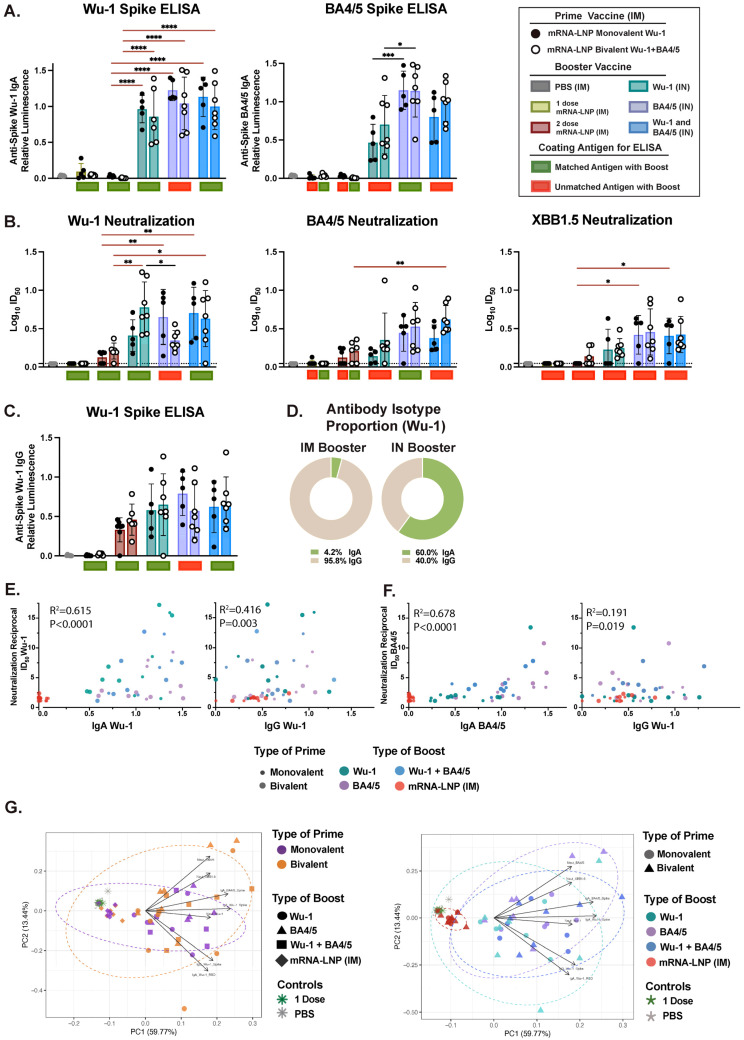
Characterization of mucosal immune responses in bronchoalveolar lavage fluid (BALF) following a heterologous systemic prime and mucosal boost vaccination. (**A**) Measurement of Wu-1- and BA4/5-spike-specific IgA in BALF at endpoint (day 35). (**B**) Reciprocal ID₅₀ neutralization titers based on inhibition of soluble ACE2 binding to immobilized Wu-1, BA.4/5, and XBB.1.5 spike proteins. (**C**) Measurement of Wu-1-spike-specific IgG titers. (**D**) Relative proportion of Wu-1-specific IgG and IgA isotypes in BALF. (**E**,**F**) Correlations between (**E**) Wu-1 and (**F**) BA.4/5 neutralization (ID₅₀) titers and IgG/IgA isotypes, with data points colored by booster type. (**G**) Principal component analysis (PCA) of antibody responses in BALF. Each point represents an individual mouse, incorporating all measured antibody responses and dotted ellipses represent confidence interval for each group. PCA plots are colored by (i) prime vaccine type and (ii) booster type to illustrate distinct clustering patterns driven by different vaccination regimens. Data are from one biological replicate and represent the average of three technical replicates and dotted line represents neutralization assay cutoff. BALF was diluted 1:30 for IgA titer measurements. Antibody titers were log-transformed and analyzed using one-way ANOVA with Tukey’s multiple comparisons test. **** *p* ≤ 0.0001, *** *p* ≤ 0.0001, ** *p* ≤ 0.01, * *p* ≤ 0.05.

IM administration typically favors Ig class switching to IgG over IgA. However, stimulating the respiratory mucosa through vaccination or infection has been shown to induce robust production of IgA locally or in other distinct mucosal compartments. Indeed, the IN boost, but not the IM boost, induced robust mucosal Ig responses as detected in BAL and nasal biofluids, which predominantly favored IgA. We observed that the antigen dose administered via the IN route was important, as total Ig and neutralizing responses were significantly lower following a 1 μg dose of unadjuvanted protein IN boost relative to the 10 μg dose, which effectively boosted both IgG and IgA levels (Figure 3 and Supplementary Figure S5). We found that an IN boost induced comparable Wu-1-specific IgA levels across the differentially (matched or mismatched) boosted mice, which was significantly higher than those observed after two doses of mRNA-LNP administered IM (Figure 3A). However, we detected significantly greater BA4/5-specific IgA levels in mice IN-boosted with the BA4/5 (matched) and, although not statistically significant, with Wu-1 + BA4/5 antigen (matched), than with the Wu-1 antigen (mismatched) boost. While we observed an overall positive correlation between BA.4/5- and Wu-1-specific IgA antibodies when all IN-boosted mice were analyzed together (r = 0.837, *p* ≤ 0.0001) (Supplementary Figure S2A), this correlation was only positive in groups boosted with BA.4/5 (r = 0.87, *p* = 0.001) and Wu-1 + BA.4/5 (r = 0.87, *p* = 0.003) antigens. No correlation was observed in the Wu-1-boosted group (r = 0.20, *p* = 0.543). In general, in contrast to differences in BA4/5-specific IgG levels between monovalently and bivalently primed mice observed in serum, differences in BA4/5-specific IgA levels were influenced by the vaccine formulation of the boost rather than the prime.

Next, we evaluated spike neutralizing activity in BALF and found detectable but weak neutralization activity in mice that were IN-boosted with the 10 μg dose of unadjuvanted protein (Figure 3B). Consistent with systemic neutralizing responses, spike–ACE2 binding inhibition for specific variants was superior in mice boosted with the corresponding (matched) or closely related antigen. In line with the serological data, we observed comparable Wu-1 inhibition in mice across differentially IN-boosted mice and was notably higher than that observed following two doses of mRNA-LNP via IM vaccination. However, similar to the findings in serum, mice primed with the bivalent mRNA-LNP vaccine and boosted with BA4/5 antigen (mismatched) exhibited significantly lower Wu-1 spike inhibition. Generally, an IN boost with BA4/5 + Wu-1 antigen (matched) elicited enhanced BA4/5 and XBB1.5 spike neutralization, which was significantly higher than that observed in mice IM-boosted with the monovalent mRNA-LNP. Given that mucosal IgA has been shown to effectively inhibit viral infection in vitro across both animal models and human studies [23,66,73,76,80,86], we sought to determine the contribution of IgA relative to IgG in mucosal neutralization activity in vitro. We found that IgA antibody titers positively correlated with spike neutralizing activity against Wu-1 (r = 0.615, *p* ≤ 0.0001) and BA4/5 (r = 0.678, *p* ≤ 0.0001) compared to IgG (Figure 3E,F). Furthermore, no correlation was observed between Wu-1 and BA4/5 or BA4/5 and XBB1.5 neutralization (Supplementary Figure S2B,C). Indeed, we observed distinct clustering based on the route of administration in the PCA and dose of the IN booster, where the 1 μg IN- and IM-boosted mice grouped independently from the 10 μg-boosted mice (Figure 3G and Supplementary Figure S5E). Taken together, these findings suggest that in contrast to serum, where the prime vaccination overwhelmingly dictated the humoral response outcome, humoral responses in the BALF were predominantly influenced by the booster formulation, with antigen-specific IgA response as the primary driver.

**Figure 4:**
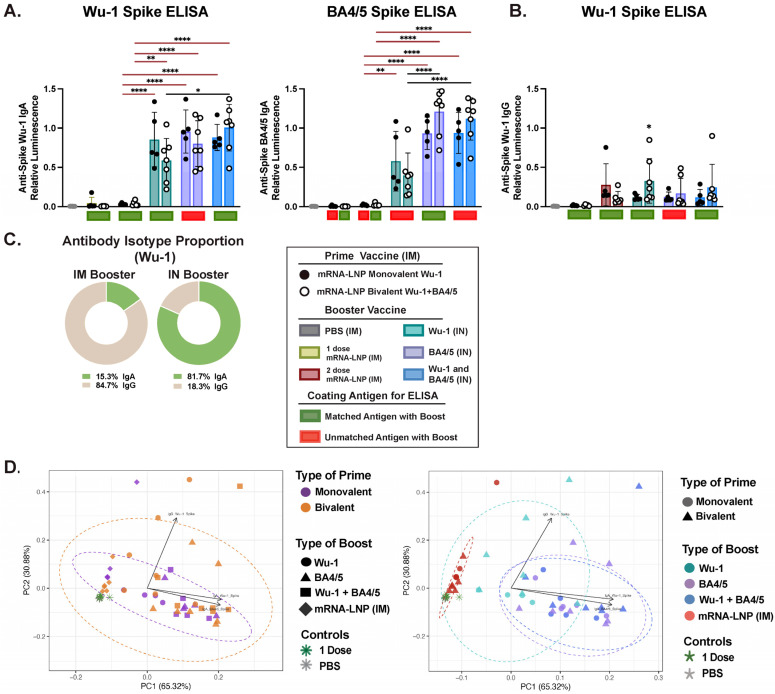
Characterization of mucosal immune responses in nasal wash following a heterologous systemic prime and mucosal boost vaccination. (**A**) Measurement of Wu-1- and BA4/5-spike-specific IgA in nasal wash at endpoint (day 35). (**B**) Measurement of Wu-1-spike-specific IgG titers and (**C**) relative proportion of Wu-1-specific IgG and IgA isotypes in nasal wash. (**D**) Principal component analysis (PCA) of antibody responses in nasal wash and dotted ellipses represent confidence interval for each group. Each point represents an individual mouse, incorporating all measured antibody responses. PCA plots are colored by (i) prime vaccine type and (ii) booster type to illustrate distinct clustering patterns driven by different vaccination regimens. Data reflect results obtained from one biological replicate and are representative as average of 2–3 technical replicates. Nasal wash was diluted 1:5 for the measure of IgA-specific titers. For statistical analysis, antibody titers were log-transformed and then analyzed by a one-way ANOVA with Tukey’s multiple comparisons. **** *p* ≤ 0.0001, ** *p* ≤ 0.01, * *p* ≤ 0.05.

**Figure 5:**
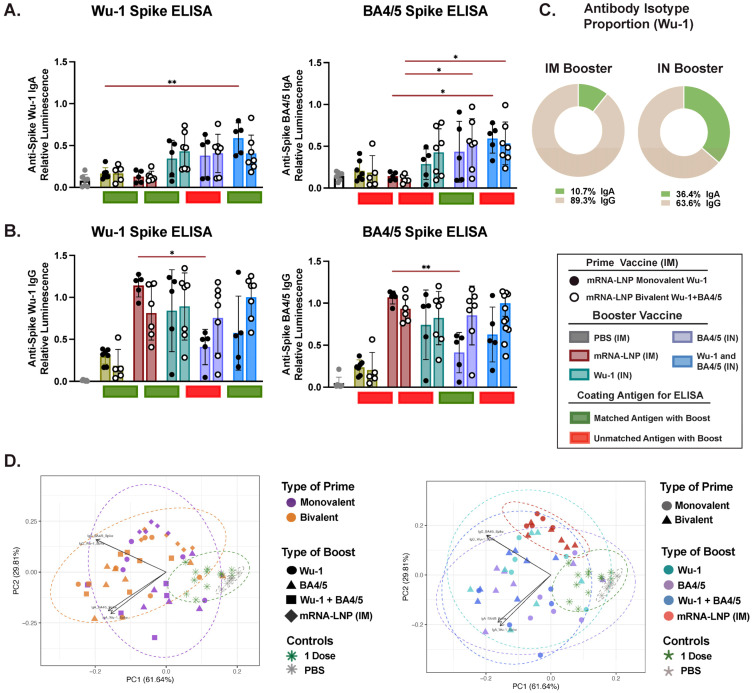
Characterization of mucosal immune responses in intestines following a heterologous systemic prime and mucosal boost vaccination. (**A**) Measurement of Wu-1- and BA4/5-spike-specific IgA titers in intestinal fluid at endpoint (day 35). (**B**) Measurement of Wu-1- and BA4/5-spike-specific IgG titers. (**C**) Relative proportion of Wu-1-specific IgG and IgA isotypes in intestinal fluid. (**D**) Principal component analysis (PCA) of antibody responses in nasal wash. Each point represents an individual mouse, incorporating all measured antibody responses and dotted ellipses represent confidence interval for each group. PCA plots are colored by (i) prime vaccine type and (ii) booster type to illustrate distinct clustering patterns driven by different vaccination regimens. Data reflect results obtained from one biological replicate and are representative as average of 2–3 technical replicates. Intestinal supernatant was diluted 1:2 for the measure of IgA-specific titers. For statistical analysis, antibody titers were log-transformed and then analyzed by a one-way ANOVA with Tukey’s multiple comparisons. ** *p* ≤ 0.01, * *p* ≤ 0.05.

Next, we assessed mucosal IgG and IgA levels in the nasal biofluid (Figure 4A and Supplementary Figure S6). Only mice that received the 10 μg IN boost developed high levels of IgA, which were significantly greater than in those that received two doses of mRNA-LNP via IM. Notably, as in BALF, a similar pattern emerged in variant-specific IgA responses, with groups boosted with the BA4/5 or BA4.5 + Wu-1 exhibiting significantly higher BA4/5-specific IgA titers. We found a strong correlation (r = 0.81, *p* ≤ 0.001) between BA4/5- and Wu-1-specific IgA antibodies, but only in the Wu-1 + BA4/5-boosted mice (Supplementary Figure S2F). Both IM mRNA-LNP and IN protein vaccinations elicited comparable IgG responses, which were detectable but not significantly higher than the PBS control group (Figure 5B). Furthermore, while the 1 μg dose IN boost elicited detectable IgA levels in the nasal biofluid, these levels were not significantly higher in all IN-boosted groups compared to the PBS control (Supplementary Figure S6A,B). Interestingly, we observed differential clustering in nasal biofluid compared to both serum and BALF, with the IM-boosted group clustering independently from both the 1 μg dose and the 10 μg dose IN boost in the PCA (Figure 4D and Supplementary Figure S6E). Furthermore, distinct clustering was observed based on the type of booster, which was primarily driven by antigen-specific IgA response. Given the Th2-skewed immune response in BALB/c mice, vaccine formulations should also be evaluated in Th1-prone strains, such as C57BL/6, to ensure a comprehensive assessment of immune responses [87,88].

### 3.4. IM and IN Vaccination Induce Potent IgG Responses in the Intestinal Lumen

**Figure 6:**
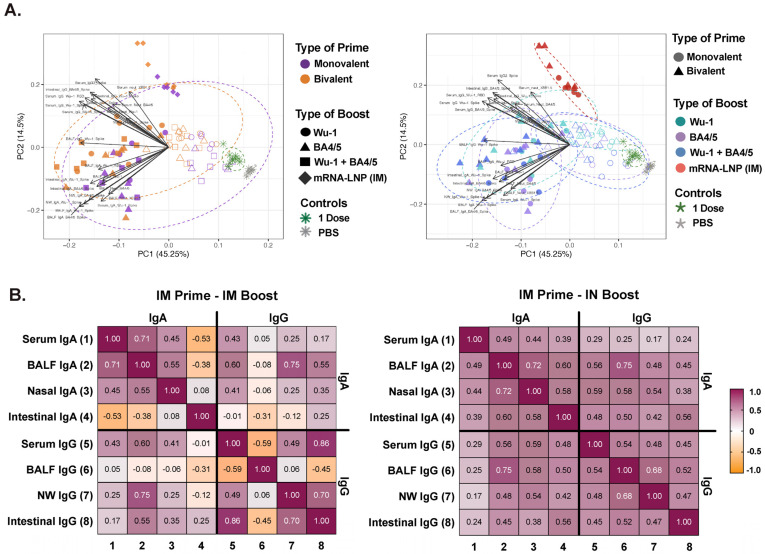
Humoral response kinetics and analysis of IgA and IgG antibodies from different compartments. (**A**) Principal component analysis (PCA) of antibody responses across serum, BALF, nasal wash, and intestinal fluid. Each point represents an individual mouse, incorporating all antibody measurements across compartments and dotted ellipses represent confidence interval for each group. PCA plots are colored by prime and boost type to illustrate differential clustering patterns based on vaccination regimens. (**B**) Correlation between IgG and IgA antibody levels in serum, BALF, nasal wash, and intestinal fluid. Each square represents total antibody responses from all mice combined, categorized by intranasal (IN) boost formulation (1 µg or 10 µg dose) or IM mRNA-LNP boost.

A single dose of mRNA-LNP administered IM induced detectable levels of IgG antibodies in the intestinal fluid (Figure 6 and Supplementary Figure S7). While IN administration of a 10 μg dose of unadjuvanted protein boosted both IgG and IgA levels, the 1 μg dose or IM administration of the mRNA-LNP vaccine only boosted IgG responses (Figure 5A,B; Supplementary Figure S7A,B). Notably, IM administration induced IgG levels that were either superior to or comparable with those elicited by the 10 μg IN boost. However, unlike in other mucosal compartments, both routes of administration elicited an IgG-predominant response in the intestinal fluid (Figure 5C and Supplementary Figure S7C). Furthermore, no variant-specific IgG- or IgA-skewed response corresponding to the type of prime or boost vaccine formulation was observed, which contrasts to findings in other compartments (Supplementary Figure S2G). We observed differential clustering based on the administration route and number of vaccine doses received, which were primarily driven by both IgG and IgA responses (Figure 5D and Supplementary Figure S7D).

### 3.5. Effect of Administration Route on Humoral Response Profiles and Antibody Correlations Across Compartments

Next, we sought to further dissect the relative influence of each administration route and vaccine formulation on the overall humoral response outcome. For this analysis, we included paired serological data from each distinct compartment across the various prime and boost groups. Notably, we observed five separate clusters primarily driving IgG and IgA responses (Figure 6A). The mRNA-LNP IM-boosted group clustered independently from the 10 μg and 1 μg protein IN-boosted groups, and was driving predominantly IgG responses, while the 1 μg dose groups clustered more closely to the 10 μg-boosted groups, with the IgA response as the primary driver. Furthermore, we additionally noted differential clustering based on the type of prime, similar to our observation in the serum. Several groups have previously reported strong to moderate IgG correlation between paired serum and saliva in convalescent COVID-19 patients, while IgA responses exhibited weaker correlation, highlighting possible compartmentalization of the IgA response [23,89,90,91].

Next, we analyzed the correlation between IgG and IgA responses across three anatomical compartments to better understand the distribution and coordination of the induced immune responses, determining whether they are compartmentalized or consistently aligned across the mucosal compartments (Figure 6B). Paired serological data from each mouse were analyzed, with antibody profiles stratified by administration route to reflect the distinct clustering observed between IN- and IM-boosted groups in Figure 6A. Of the three mucosal compartments, we observed high IgA (r = 0.72, *p* ≤ 0.0001) and IgG (r = 0.68, *p* ≤ 0.0001) correlation between BALF and nasal wash in IN-boosted mice only. While a positive correlation (r = 0.75, *p* ≤ 0.0001) between IgG and IgA responses was observed in BALF in IN-boosted mice, no correlation between these antibody isotypes in other biofluids was observed. While a positive IgA correlation (r = 71, *p* = 0.008) was observed between serum and BALF in IM-boosted mice, it was likely confounded given the very low IgA titers detected in BALF following IM vaccination. Interestingly, we found a strong positive correlation (r = 0.86, *p* = 0.001) between serum and intestinal IgG antibody titers in IM-boosted mice levels, which was not observed in IN-boosted mice.

## 4. Discussion

In this study, we aimed to examine the effects of heterologous prime–boost regimens on humoral responses, focusing on IM and IN administration routes and variant-tailored vaccine formulations. Our findings indicate that the IM prime predominantly shapes systemic humoral responses, primarily driven by variant-specific neutralizing responses, while the IN boost further amplifies and refines them. Indeed, homologous prime and boost antigen vaccination resulted in Wu-1-skewed humoral responses. This effect was mitigated by heterologous IN boosting with the Omicron BA4/5 antigen, which significantly improved neutralization against antigenically distant VOCs compared to mice that received two doses of the monovalent mRNA-LNP IM vaccine, while retaining neutralization activity against the ancestral Wu-1 strain. Importantly, our results highlight the value of incorporating distinct antigenic variants in the prime vaccine, as the bivalent prime significantly enhanced the breadth of total and neutralizing responses against circulating VOCs in serum. The IN boost, rather than the IM prime, had a greater impact on mucosal responses, primarily driven by antigen-specific IgA responses in nasal wash and BALF. Higher IgA titers correlated more strongly with neutralizing activity than IgG, with the BA4/5- and BA4/5 + Wu-1-boosted groups showing the highest neutralizing titers against BA4/5. Furthermore, we show differential induction of IgG and IgA antibody isotypes across distinct immune compartments following IM relative to IN boosting. Indeed, a strong IgA antibody correlation between mucosal compartments was observed only in IN-vaccinated mice. Taken together, our findings highlight the merit of including distinct antigenic variants, if possible, in the prime vaccine to enhance VOC neutralization and support the use of variant-tailored IN boosters, which simultaneously boost systemic responses and elicit mucosal responses at the viral point of entry.

Additionally, we demonstrate that IN was significantly superior to IM vaccination at driving a predominantly IgA-favored response in nasal, BAL, and intestinal biofluids. This observation aligns with recent studies evaluating heterologous prime–boost regimens in vivo, which highlight the potent induction of cellular and humoral responses in BALF following IN or oral vaccination in mice with pre-existing immunity [22,70,72,73,74,75,79,80]. However, while variant-specific total and neutralizing responses after variant-tailored boosting are well documented in serum, the effects of heterologous boosting strategies on the induction of variant-specific responses in saliva, nasal, and BAL biofluids remain poorly characterized. Here, we demonstrate that variant-specific IgA responses in BALF and nasal wash were predominantly shaped by the vaccine antigens delivered via IN rather than IM immunization, highlighting the compartmentalization of mucosal responses as recently suggested [23,89,90,91]. Given that transmission of SARS-CoV-2 primarily occurs via inhalation of aerosolized virus, local immune responses are uniquely positioned to act as a rapid, first line of defense against infection. Initial mRNA-LNP IM vaccines were up to 90% effective against symptomatic infection following exposure to antigenically matched VOCs [15,16,17,29], likely due to potent induction of systemic IgG response, which could diffuse into the URT [56,92]. Our findings of robust IgG titers in BALF and nasal wash following the mRNA-LNP boost support this. However, Omicron and its sub-variants evolved to preferentially replicate in the URT and have shorter incubation times and thereby reduce the immune recall response window [93,94,95]. This leads to increased viral transmissibility and emphasizes the need for a persistent virus-neutralizing capacity at the initial replication site.

A growing body of literature highlights the importance of stimulating the respiratory mucosa and the role of sIgA in respiratory mucosa to protect against breakthrough infections [22,23,43,55,56,57,58,59,96]. Despite similar systemic IgG levels, individuals with hybrid immunity (vaccination plus infection) exhibit higher mucosal IgA levels, which were associated with a lower incidence of subsequent breakthrough infections compared to vaccinated-only individuals [22,43,55,56,57,58]. We found that IgA responses in BALF correlated more strongly with neutralizing activity than the IgG isotype, consistent with studies showing sIgA’s potent neutralization capacity against SARS-CoV-2 in vitro [23,66,73,76,80,86]. Notably, we observed comparable BA4/5- and XBB1.5-neutralizing activity in BALF, suggesting that IgA, abundant in dimeric or pentameric forms at the mucosal surface, may be more effective than monomeric IgG at neutralizing variants with escape [86,97]. While systemic neutralizing antibodies are an established correlate of vaccine protection [83], analogous correlates of mucosal immunity against viral infection and transmission have yet to be established. Identifying these will be key to assessing the efficacy of intranasal vaccines. Indeed, antibody kinetics at distinct mucosal sites in response to different vaccination regimens remain poorly characterized. In this study, we found a strong correlation between IgA in BAL and nasal compartments only in IN-vaccinated mice, suggesting IgA compartmentalization, as observed in studies assessing IgA correlations in serum and saliva [23,89,90,91].

Recent evidence shows that SARS-CoV-2 breakthrough infections induce durable tissue-resident memory T and B cells, including plasma and germinal center B cells, persisting for up to a year in humans, with IgA^+^ memory B cells significantly enriched in the upper airways compared to blood [98]. IgA is the predominant immunoglobulin at mucosal surfaces, while IgG, though less abundant, can constitute 15–20% of total immunoglobulins at certain sites [99,100] and plays a protective role in the lower respiratory tract through complement activation and antibody-dependent cellular cytotoxicity (ADCC) [92,101]. In serum, IgA exists primarily in monomeric form, accounting for approximately 15% of total immunoglobulins [102,103], contributing to immune defense via phagocytosis, ADCC, and, under specific conditions, complement activation. Building on these observations, our study offers a comprehensive comparison of relative IgA and IgG levels in serum and across distinct anatomical compartments following IN or IM SARS-CoV-2 vaccination. This comparison is crucial for understanding how different vaccination routes shape antibody distribution across compartments and contribute to mucosal and systemic immunity, informing strategies for optimizing vaccine efficacy.

Interestingly, we observed IgG responses in the intestinal mucosa following both IM and IN immunization. While plasma cells can disseminate between distinct mucosal compartments after mucosal immunization [96,104,105], it remains unclear how IgG responses were generated after IM vaccination. Spike protein has been detected in plasma for up to five days following mRNA-LNP IM vaccination [106], suggesting that these antibody responses might be induced directly in Peyer’s patches, which are present in the intestinal tissue we analyzed. Notably, residual SARS-CoV-2 antigens have also been detected in intestinal mucosa and stool samples, even after viral clearance from the URT [107,108,109]. Thus, this potent induction of humoral responses in the intestinal lumen following IM vaccination with mRNA-LNP should be studied and potentially leveraged to reduce viral replication and transmission of gastrointestinal viruses.

We observed reduced BA4/5 and XBB.1.5 neutralization in mice primed with the monovalent Wu-1 vaccine and boosted with the BA4/5 + Wu-1 combination, compared to those boosted with BA.4/5 antigen alone. In contrast, mice bivalently primed and boosted with either BA.4/5 alone or the BA.4/5 + Wu-1 combination showed comparable neutralization activity across both formulations. These findings suggest that the BA4/5 + Wu-1 boost in monovalent-primed mice primarily triggered cross-reactive recall responses to shared epitopes, rather than inducing BA.4/5-specific antibodies, indicating the presence of immune imprinting. This aligns with recent studies showing that variant-specific boosting primarily promoted recall responses, with the highest neutralizing titers against the ancestral strain [4,20,38,41,42,50,51,52,53]. This immune imprinting effect was further exacerbated by retaining the ancestral antigen in the bivalent vaccines. The generation of variant-specific de novo antibodies has been observed, although at low levels, with monovalent Omicron- and XBB1.5-specific mRNA-LNP boosters, highlighting the benefits of updating vaccines to match circulating variants [41,42,110,111]. However, these findings highlight the influence of immune imprinting, which can be overcome by boosting with antigens that are sufficiently distinct from the priming antigen [110], through repeated boosting [112], or by incorporating adjuvants [113], as we have previously reviewed [48].

Early after SARS-CoV-2 infection, cellular immune responses are crucial in controlling infection severity and providing long-term protection, while vaccine-elicited total and neutralizing antibody responses are more closely associated with protection against infection and symptomatic disease [83,84]. Given that most T cell epitopes remain relatively unchanged in VOCs [114], and that cellular responses were characterized in detail in the “Prime and Spike” vaccination approach by Mao et al., this study focused exclusively on examining only the humoral antibody profiles across different anatomical compartments using monovalent and bivalent vaccines.

Our findings demonstrate that bivalent priming with two distinct variants significantly broadened the neutralization responses, which can be further enhanced by IN boosting. The inclusion of multivalent viral strains or a highly conserved antigen in vaccine formulations, as proposed for some universal vaccine strategies, can leverage immune imprinting to shape a broadly reactive B cell repertoire. This approach may be particularly beneficial for immunologically naïve populations, such as young children, by providing broad protection against circulating SARS-CoV-2 variants. Additionally, it could be an effective strategy for combating future emerging respiratory viruses before widespread exposure occurs. Understanding the impact of different vaccination strategies on immune responses across different anatomical compartments may aid in inducing protective mucosal responses against SARS-CoV-2 variants.

## 5. Conclusions

Our study showcases the benefits of heterologous prime–boost vaccination strategies, particularly the role of IN boosting in enhancing both systemic and mucosal immunity against SARS-CoV-2 variants. We demonstrate that an IM prime predominantly drives systemic IgG responses, while an IN boost significantly amplifies variant-specific neutralization and elicits robust mucosal IgA responses, which correlate strongly with neutralization activity in the respiratory tract. Importantly, incorporating antigenically distinct variants in the prime vaccine broadens humoral responses and mitigates immune imprinting. Our findings support the inclusion of variant-adapted IN boosters to enhance protection at the viral entry site, highlighting their potential in mitigating breakthrough infections and transmission. Understanding the differential impact of IM and IN vaccination across immune compartments will be crucial for optimizing next-generation vaccine strategies against SARS-CoV-2 and other respiratory pathogens.

## Figures and Tables

**Table 1:** Vaccination regimens administered to mice.

Mode of Administration (Prime–Boost) *	Type of Vaccine Antigenic Formulation	Prime (Day 1)	Boost (Day 21)
IM–IN	-	PBS	PBS
IM–IN	-	Monovalent mRNA-LNP	PBS
IM–IN	-	Bivalent mRNA-LNP	PBS
IN–IN	Homologous	Wu-1 protein	Wu-1 protein
IM–IM	Homologous	Monovalent mRNA-LNP	Monovalent mRNA-LNP
IM–IN	Homologous	Monovalent mRNA-LNP	Wu-1 protein
IM–IN	Heterologous	Monovalent mRNA-LNP	BA4/5 protein
IM–IN	Heterologous	Monovalent mRNA-LNP	Wu-1 + BA4/5 protein
IM–IM	Homologous	Bivalent mRNA-LNP	Bivalent mRNA-LNP
IM–IN	Homologous	Bivalent mRNA-LNP	Wu-1 protein
IM–IN	Homologous	Bivalent mRNA-LNP	BA4/5 protein
IM–IN	Homologous	Bivalent mRNA-LNP	Wu-1 + BA4/5 protein
IM–IN	Heterologous	Bivalent mRNA-LNP	NL63 protein

* IM: intramuscular LNP-mRNA Monovalent: Pfizer-Comirnaty monovalent Wuhan (Wu-1) or bivalent (Wu-1 + BA.4/5) (1 µg dose of total vaccine); IN: intranasal unadjuvanted spike protein as indicated (1 µg or 10 µg doses of total antigen).

## Data Availability

The raw data supporting the conclusions of this article will be made available by the authors on request.

## Supplementary Materials

The following supporting information can be downloaded at: https://www.mdpi.com/article/10.3390/vaccines13040351/s1, Figure S1: Prime vaccination induces distinct humoral responses, while booster vaccination further refines total and neutralizing antibody profiles in serum; Figure S2: Dissection of mucosal immune responses across distinct biofluids; Figure S3: Characterization of systemic immune responses in serum following a heterologous systemic prime and 1 μg mucosal boost vaccination. Figure S4: Kinetics of systemic immune responses in serum following a heterologous systemic prime and 1 μg mucosal boost vaccination. Figure S5: Characterization of mucosal immune responses in BALF following a heterologous systemic prime and 1 μg mucosal boost vaccination. Figure S6: Characterization of mucosal immune responses in nasal fluid following a heterologous systemic prime and 1 μg mucosal boost vaccination; Figure S7: Characterization of mucosal immune responses in intestinal fluid following a heterologous systemic prime and 1 μg mucosal boost vaccination.
