# Supplementary material for: Systemic and Mucosal Antibody Responses to SARS-CoV-2 Variant-Specific Prime-and-Boost and Prime-and-Spike Vaccination: A Comparison of Intramuscular and Intranasal Bivalent Vaccine Administration in a Murine Model
Source: Vaccines (Basel). 2025 Mar 25;13(4):351. doi: 10.3390/vaccines13040351 (PMC12031244; doi:10.3390/vaccines13040351)
Supplement: Supplementary file 1 [file vaccines-13-00351-s001.zip › vaccines-3532191-supplementary.pdf]

SUPPLEMENTARY MATERIALS

Systemic and Mucosal Antibody Responses to SARS-CoV-2 Variant-Specific Prime-and-Boost and Prime-and-Spike Vaccination: A Comparison of Intramuscular and Intranasal Bivalent Vaccine Administration in a Murine Model

Mariam Maltseva[1], Yannick Galipeau[1], Pauline McCluskie[1], Nicolas Castonguay[1], Curtis L. Cooper[2], and Marc-André Langlois[1,3]

[1]Department of Microbiology and Immunology, Faculty of Medicine, University of Ottawa, Ottawa, Ontario, Canada.

[2]The Ottawa Hospital Research institute

[3] University of Ottawa Ottawa Center for Infection, Immunity, and Inflammation (CI3).

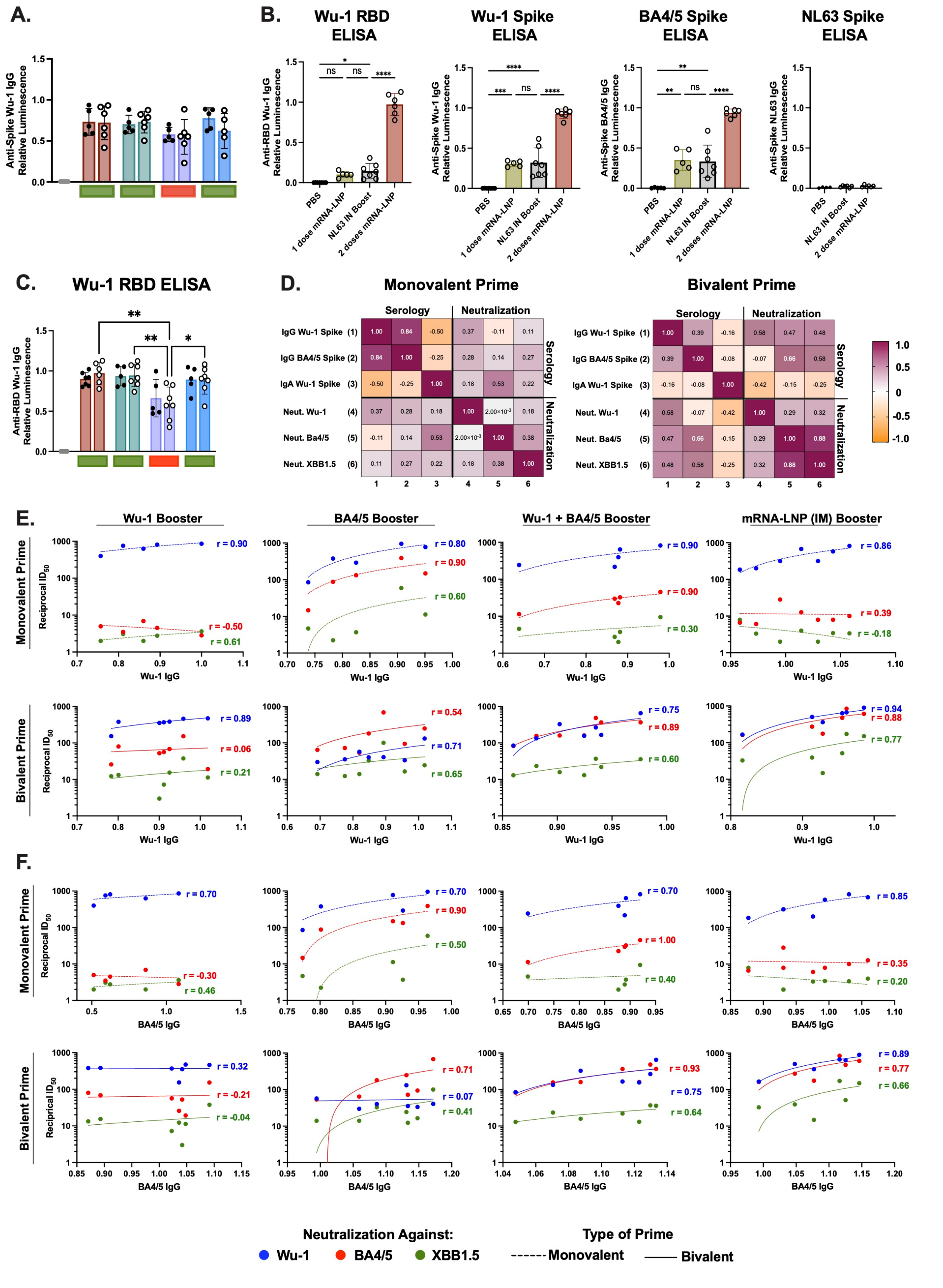

Supplementary Figure S1. Prime vaccination induces distinct humoral responses, while booster vaccination further refines total and neutralizing antibody profiles in serum. A) Wu-1 spike-specific IgG titers in serum collected before the second dose administration. **B)** Wu-1, BA.4/5, and NL63 spike-specific IgG titers in serum at the study endpoint for mice intranasally boosted with a 10 µg dose of NL63 seasonal coronavirus protein. **C)** Wu-1 receptor-binding domain (RBD)-specific IgG titers in serum at the

study endpoint for mice receiving an NL63 protein boost. **D)** Correlation between total and neutralizing antibodies in serum from monovalently or bivalently primed mice. Each square represents the combined antibody responses of all mice that received different IN boost formulations (10 µg dose) or mRNA-LNP boosts. **E-F)** Neutralization (ID_{50}) titers and Wu-1 or BA.4/5 spike-specific IgG titers in serum following different vaccination regimens. **E)** Wu-1-specific IgG and neutralization titers against Wu-1 (blue), BA.4/5 (red), and XBB.1.5 (green). **F)** BA.4/5-specific IgG and neutralization titers against Wu-1 (blue), BA.4/5 (red), and XBB.1.5 (green). Data represent one biological replicate and are shown as the average of 2–3 technical replicates. Serum was diluted 1:300 for pre-second dose evaluation and 1:500 for all subsequent IgG titer measurements. Antibody titers were log-transformed and analyzed using one-way ANOVA with Tukey's multiple comparisons test. ****$p \leq 0.0001$, **$p \leq 0.01$, *$p \leq 0.05$, n.s.: not significant.

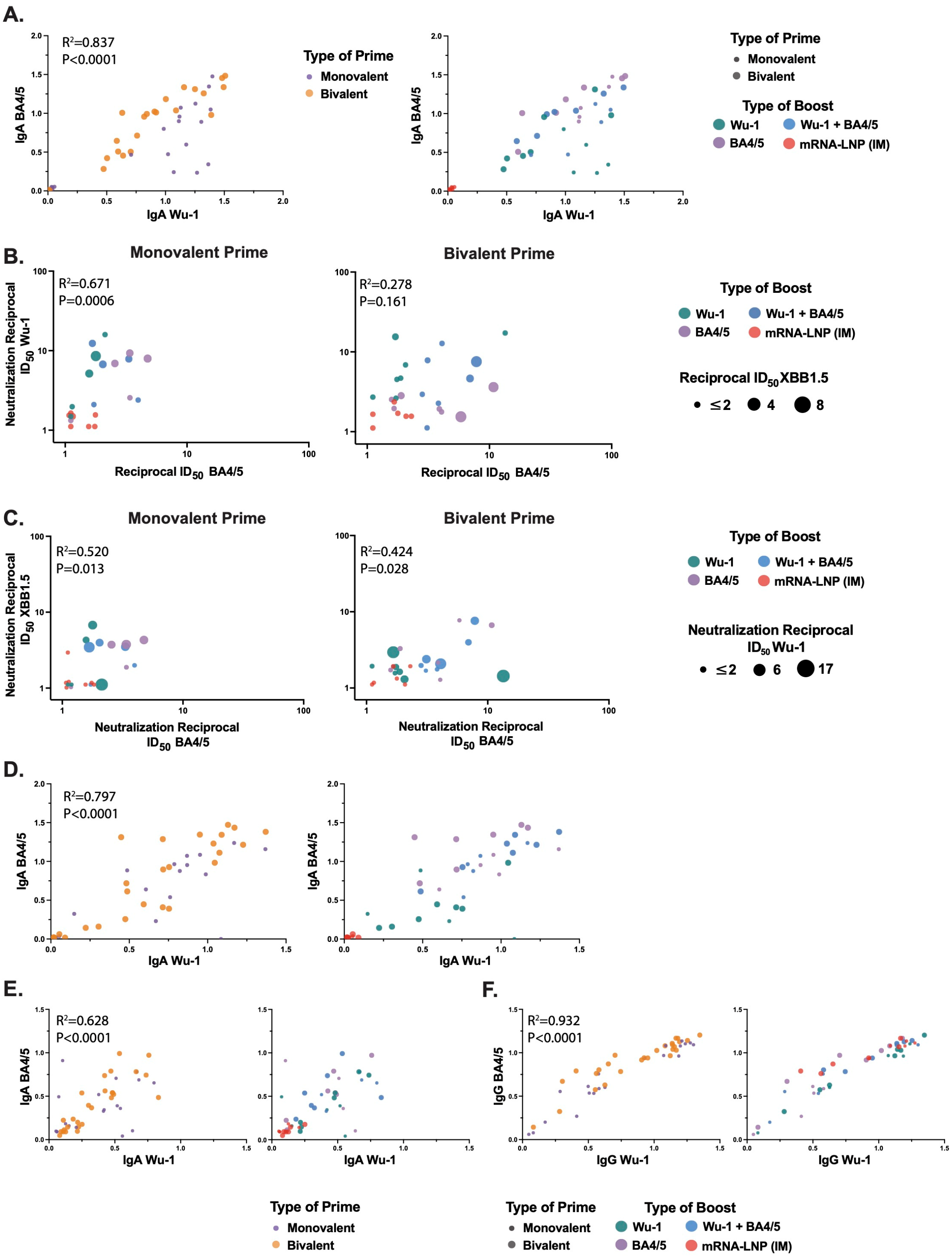

Supplementary Figure S2. Dissection of mucosal immune responses across distinct biofluids. A) Correlation between BA.4/5 and Wu-1 spike-specific IgA titers in BALF, colored by prime and boost type. **B)** Correlation between BA.4/5 and Wu-1 neutralization (ID_{50}) titers in BALF, colored by booster type. Point size reflects neutralization potency against the XBB.1.5 variant. **C)** Correlation between BA.4/5 and XBB.1.5 neutralization (ID_{50}) titers in BALF, colored by booster type. Point size reflects neutralization potency against the Wu-1 strain. **D)** Correlation between BA.4/5 and Wu-1 spike-specific IgA titers in nasal

wash, colored by prime and boost type. Correlation of BA4/5 and Wu-1 specific **E)** IgA and **F)** IgG titers in intestinal fluid colored by prime and boost type.

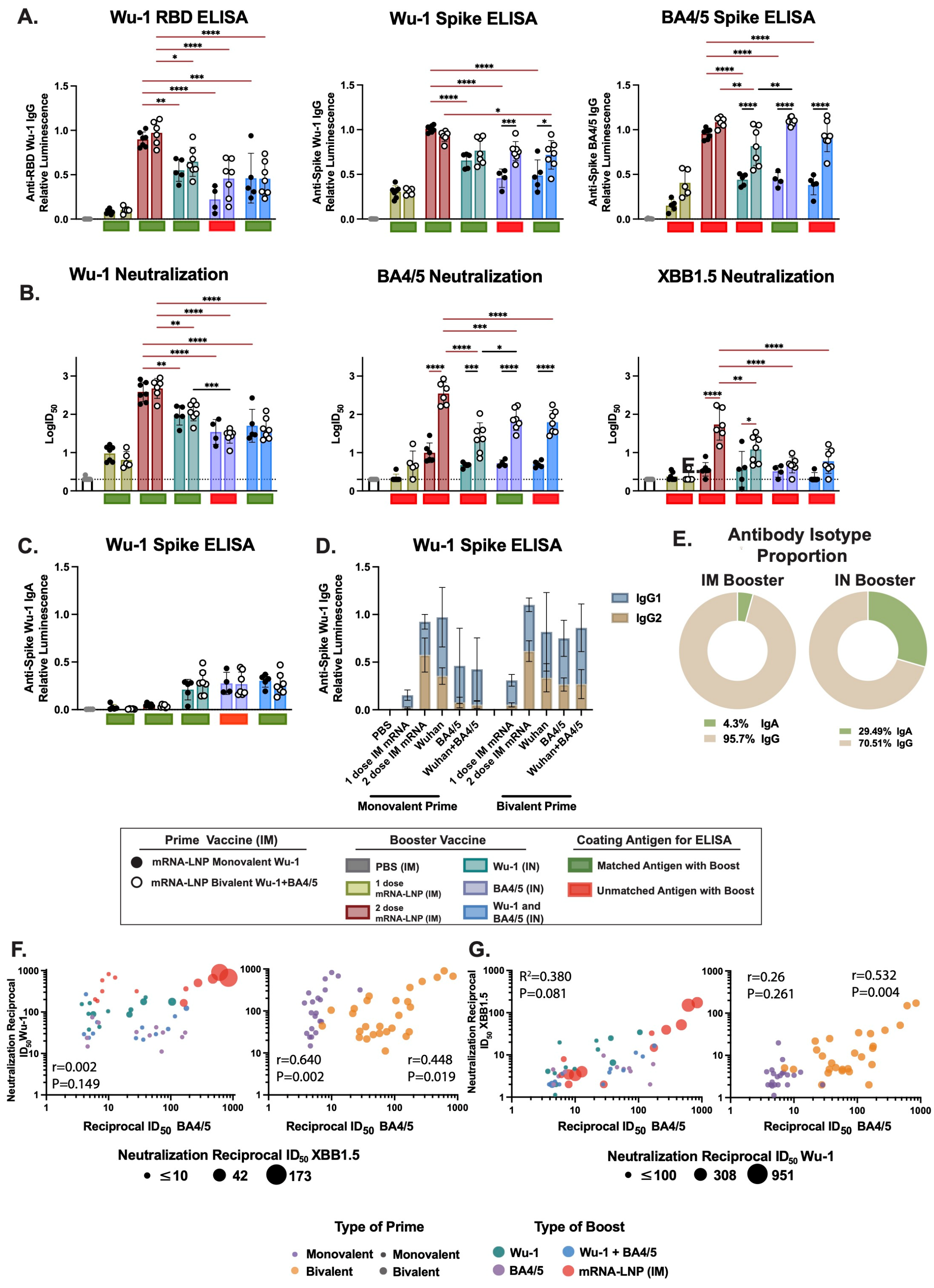

Supplementary Figure S3. Characterization of systemic immune responses in serum following a heterologous systemic prime and 1 µg mucosal boost vaccination. A) Wu-1 RBD, Wu-1 spike, and BA.4/5 spike-specific IgG titers in serum at the study endpoint. **B)** Reciprocal ID_{50} neutralization titers based on inhibition of soluble ACE2 binding to immobilized Wu-1, BA.4/5, and XBB.1.5 spike proteins. **C)** Measurement of Wu-1 spike-specific IgA in serum. **D)** IgG1 and IgG2 in serum and relative proportion

of Wu-1 specific IgG and IgA isotypes in serum. **(E)** Correlation between BA.4/5 and Wu-1 neutralization ID_{50} titers. The first graph is colored by booster type, with point size reflecting neutralization potency against XBB.1.5; the second graph presents the same data, colored by prime type. Correlations of **(F)** BA.4/5 and **(G)** XBB.1.5 neutralization ID_{50} titers. The first graph in each panel is colored by booster type, with point size reflecting neutralization potency against the Wu-1 variant, while the second graph presents the same data, colored by prime type.Data represent results from one biological replicate and are shown as the average of three technical replicates. Antibody titers were log-transformed and analyzed using one-way ANOVA with Tukey's multiple comparisons test. ****$p \leq 0.0001$, **$p \leq 0.01$, *$p \leq 0.05$, n.s.: not significant.

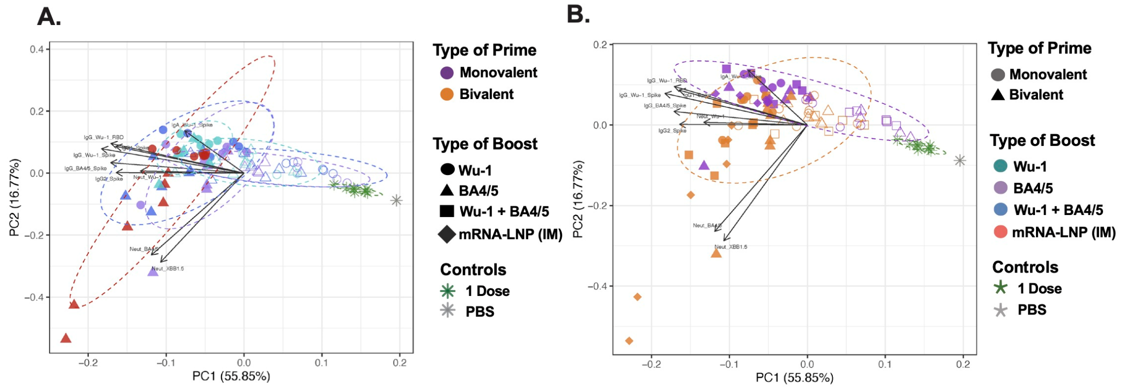

Supplementary Figure S4. Kinetics of systemic immune responses in serum following a heterologous systemic prime and 1 µg mucosal boost vaccination. Principal component analysis (PCA) of antibody responses in serum following a **10 µg** (filled points) or **1 µg** (open points) intranasal boost. Each point represents an individual mouse, incorporating all antibody measurements summarized in **Supplementary Figure 3**. PCA plots are colored by A) prime vaccine type or **B)** booster type. These analyses illustrate distinct clustering patterns of antibody responses based on different vaccination regimens.

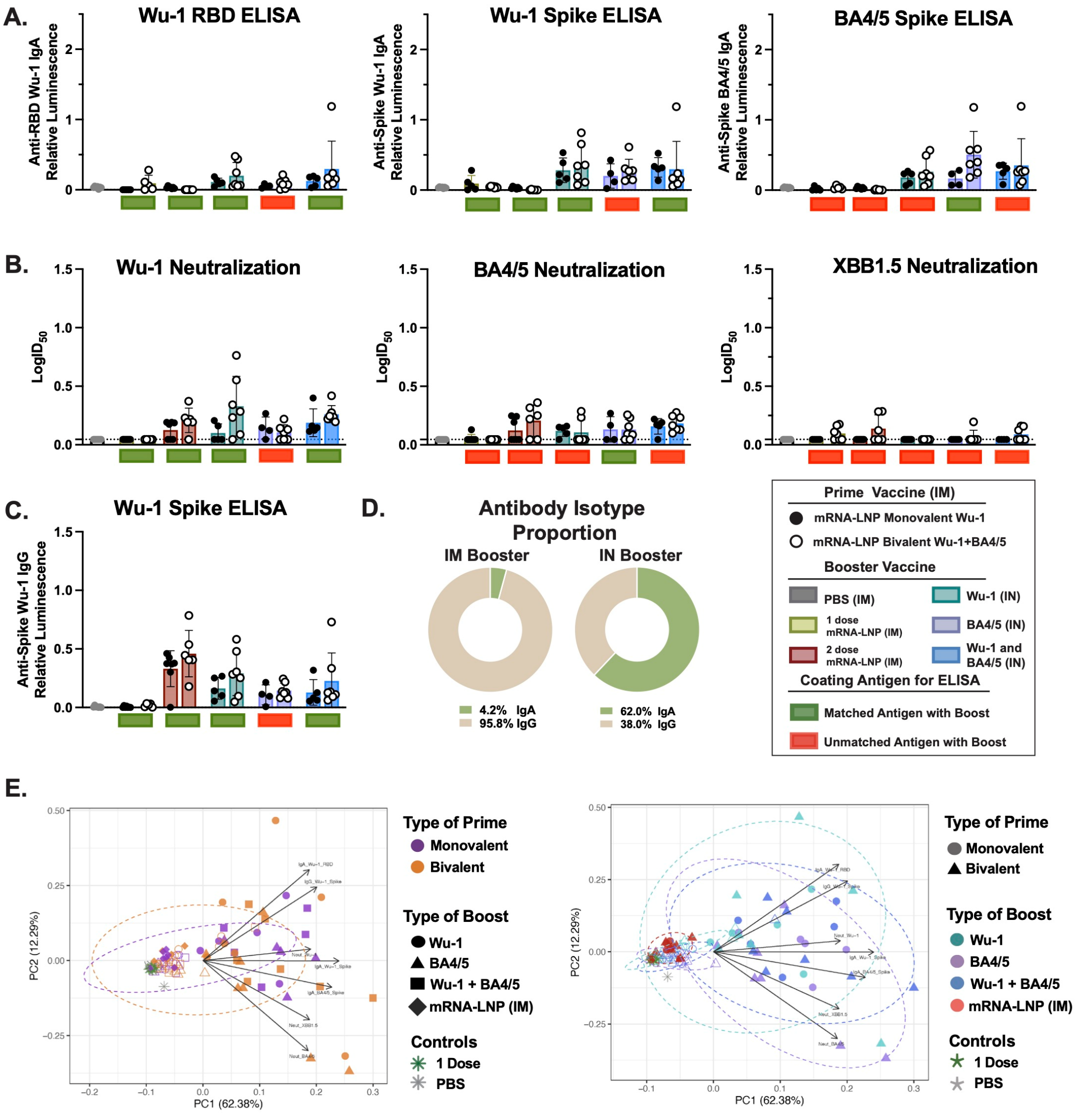

Supplementary Figure S5. Characterization of mucosal immune responses in BALF following a heterologous systemic prime and 1 µg mucosal boost vaccination. A) Wu-1 and BA.4/5 spike-specific IgA titers in BALF at the study endpoint. **B)** Reciprocal ID_{50} neutralization titers based on inhibition of soluble ACE2 binding to immobilized Wu-1, BA.4/5, and XBB.1.5 spike proteins. **C)** Wu-1 spike-specific IgG titers in BALF. **D)** Relative proportions of Wu-1 spike-specific IgG and IgA isotypes in BALF. **E)** Principal component analysis (PCA) of BALF antibody responses following a 10 µg (filled points) or 1 µg (open points) intranasal boost. Each point represents an individual mouse, incorporating all antibody measurements summarized in panels (A-C). PCA plots are colored by prime and boost type to illustrate distinct clustering patterns driven by different vaccination regimens. Data represent results from one biological replicate and are shown as the average of three technical replicates. Antibody titers were log-transformed and analyzed using one-way ANOVA with Tukey's multiple comparisons test. ****$p \leq 0.0001$, **$p \leq 0.01$, *$p \leq 0.05$, n.s.: not significant.

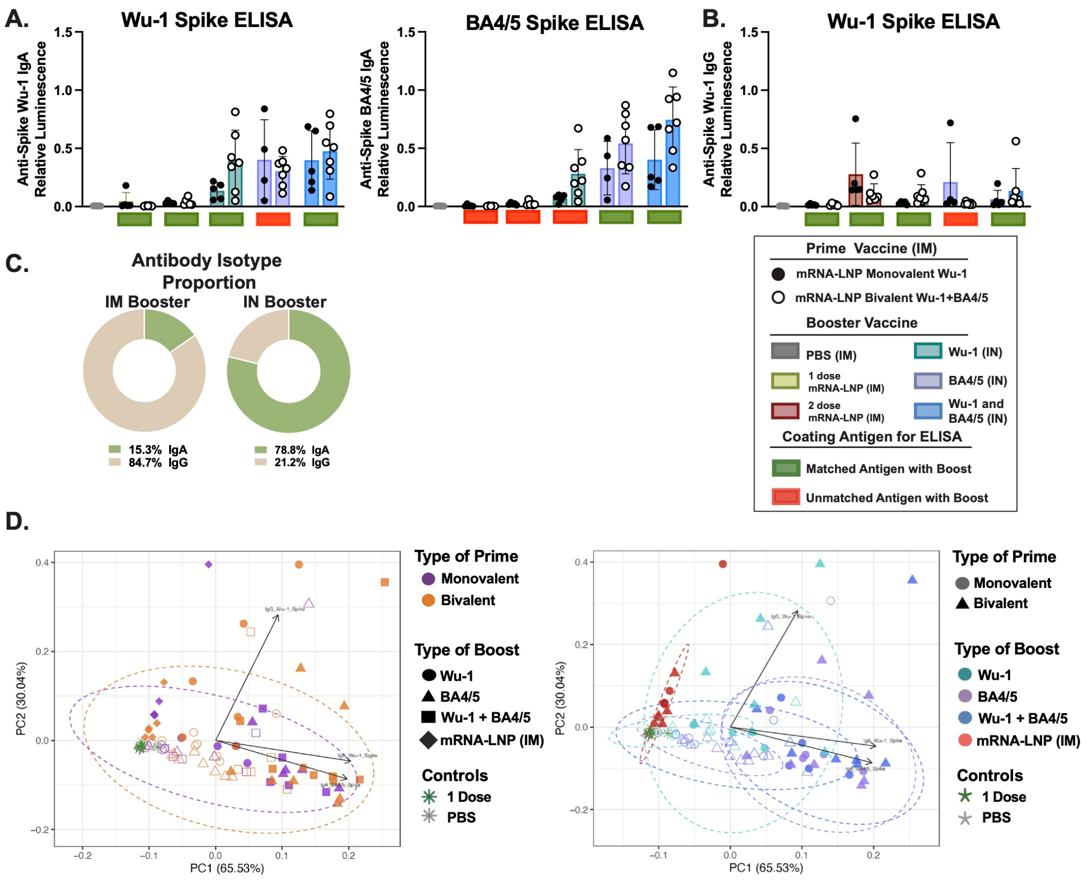

Supplementary Figure S6. Characterization of mucosal immune responses in nasal fluid following a heterologous systemic prime and 1 µg mucosal boost vaccination. A) Wu-1 and BA.4/5 spike-specific IgA titers in nasal wash at the study endpoint. **B)** Wu-1 spike-specific IgG titers in nasal wash. **C)** Relative proportions of Wu-1 spike-specific IgG and IgA isotypes in nasal wash. **D)** Principal component analysis (PCA) of antibody responses in nasal wash following a 10 µg (filled points) or 1 µg (open points) intranasal boost. Each point represents an individual mouse, incorporating all antibody measurements summarized in panels (A-C). PCA plots are colored by prime and boost type to illustrate distinct clustering patterns driven by different vaccination regimens. Data represent results from one biological replicate and are shown as the average of 2-3 technical replicates. Antibody titers were log-transformed and analyzed using one-way ANOVA with Tukey's multiple comparisons test. ****$p \leq 0.0001$, **$p \leq 0.01$, *$p \leq 0.05$, n.s.: not significant.

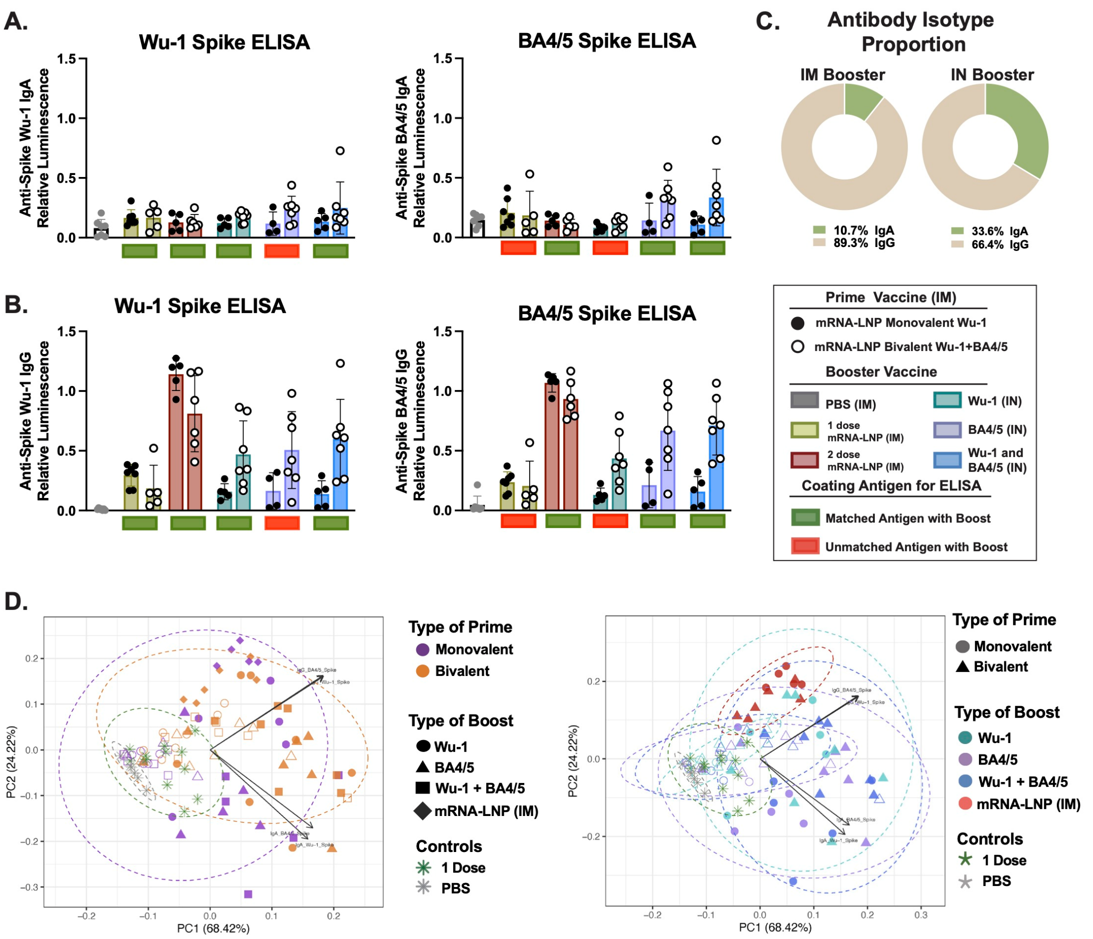

Supplementary Figure S7. Characterization of mucosal immune responses in intestinal fluid following a heterologous systemic prime and 1 µg mucosal boost vaccination. A) Measurement of Wu-1 and BA4/5 spike-specific **A)** IgA and **B)** IgG in intestines at end point. **C)** Relative proportion of Wu-1 specific IgG and IgA isotypes in serum. **(D)** Principal component analysis (PCA) of antibody responses in intestinal fluid following a **10 µg** (filled points) or **1 µg** (open points) intranasal boost. Each point represents an individual mouse, incorporating all antibody measurements summarized in panels (A-C). PCA plots are colored by prime and boost type to illustrate distinct clustering patterns driven by different vaccination regimens. Data represent results from one biological replicate and are shown as the average of three technical replicates. Antibody titers were log-transformed and analyzed using one-way ANOVA with Tukey's multiple comparisons test. ****$p \leq 0.0001$, **$p \leq 0.01$, *$p \leq 0.05$, n.s.: not significant.
